# Using Transfer Function Analysis to develop biologically and economically efficient restoration strategies

**DOI:** 10.1038/s41598-018-20178-7

**Published:** 2018-02-01

**Authors:** Lalasia Bialic-Murphy, Orou G. Gaoue, Tiffany Knight

**Affiliations:** 1 Department of Ecology and Evolutionary Biology, University of Tennessee Knoxville, Knoxville, Tennessee USA; 2 Department of Botany, University of Hawai’i at Mānoa, Honolulu, Hawai’i USA; 3 Faculty of Agronomy, University of Parakou, Parakou, Benin; 4 Department of Geography, Environmental Management and Energy Studies, University of Johannesburg, APK Campus, Johannesburg, South Africa; 5 Department of Community Ecology, Helmholtz Centre for Environmental Research-UFZ, Halle (Saale), Germany; 6 Institute of Biology/Geobotany and Botanical Garden, Martin Luther University Halle-Wittenberg, Halle (Saale), Germany; 7 German Centre for Integrative Biodiversity Research (iDiv) Halle-Jena-Leipzig, Leipzig, Germany

## Abstract

Rare species across taxonomic groups and biomes commonly suffer from multiple threats and require intensive restoration, including population reintroduction and threat control. Following reintroduction, it is necessary to identify what level of threat control is needed for species to persist over time. Population reintroduction and threat control are time intensive and costly. Thus, it is pragmatic to develop economically efficient restoration strategies. We combined transfer function analysis and economic cost analysis to evaluate the effects of biologically meaningful increases in demographic processes on the persistence of a reintroduced population of a Hawaii endemic long-lived shrub, *Delissea waianaeensis*. We show that an increase in fertility by 0.419 following the suppression of non-native rodents or an increase by 0.098 in seedling growth following the suppression of invasive molluscs would stabilize the population (i.e., *λ* = 1). Though a greater increase in fertility than seedling growth was needed for the reintroduced population to persist over time, increasing fertility by suppressing rodents was the most cost effective restoration strategy. Our study emphasizes the importance of considering the effects of large increases in plant vital rates in population projections and incorporating the economic cost of management actions in demographic models when developing restoration plans for endangered species.

## Introduction

For extremely rare species, population reintroduction and the suppression of threats are commonly used restoration strategies to prevent imminent extinction^1^. The ultimate goal of these restoration strategies are to achieve long-term populations that will persistence over time^1^. However, the long-term persistence of reintroduced populations is alarmingly low^2^. Such a low rate of persistence is likely due to the widespread occurrence of threats that are difficult to manage, such as ecosystem disturbance and invasive species. In these altered landscapes, it is essential to identify which threats need to be suppressed, following species reintroduction, to achieve the desired outcome (i.e., population growth rate *λ* ≥ 1). With limited funding for conservation and the high costs of species reintroduction and threat suppression, it is critically important to identify the most economically efficient restoration strategy.

A commonly used tool to identify which vital rate, if improved by management, would have the greatest effect on plant population dynamics is elasticity analysis^3,4^. This analytical approach is a linear approximation of the relative importance of plant vital rates on population dynamics, and is therefore an appropriate tool for assessing the effect of small changes in vital rates on population growth rate. However, the relative importance of plant vital rates on population dynamics is dependent, in part, on the magnitude of the perturbation and this relationship is often nonlinear^4–6^. Thus, elasticity analysis may lead to suboptimal conclusions when prioritizing conservation actions that have large effects on targeted vital rates. Unlike elasticity analysis, transfer function analysis accounts for the nonlinear relationship between vital rates and population growth rates and is a more appropriate approach to evaluate the influence of changes in vital rates on the population growth rate at the magnitude of interest^7–9^. An additional benefit of transfer function analysis is the possibility of applying simultaneous perturbations to multiple vital rates, which can be used to account for vital rates that are linked through trade-offs and management strategies that influence multiple vital rates^9^. For these reasons, transfer function analysis is particularly useful for evaluating conservation strategies for species threatened by multiple environmental factors.

When there are multiple combinations of management actions that would result in the desired outcome, it is also important to identify which strategy is the most cost effective. Some management actions require high upfront fixed costs (e.g., equipment costs) but low variable costs (e.g., continual ongoing maintenance) and others require low upfront costs but high variable costs. Thus, it is not always intuitive which strategy will be the most cost effective in the long-term. Though rarely used, demographic modeling provides an ideal framework to explicitly compare the economic cost of various management actions when developing restoration strategies but see,^10,11^.

Recently, we evaluated the viability of a reintroduced population of the endangered Hawaiian shrub, *Delissea waianaeensis* Lammers (Campanulaceae) that is threatened by multiple factors including non-native ungulates, plants, rodents and molluscs. Removal of non-native ungulates and invasive plant management increase the stochastic short-term transient dynamics, but are not enough for this species to persist over time (Bialic-Murphy *et al*., submitted). In the present study, we used transfer function analysis to develop economically efficient restoration strategies and identify which combination of environmental stressors need to be suppressed to ensure population persistence. Specifically, we (1) assessed the effect of changes in seedling growth and fertility on the population growth rate of *D*. *waianaeensis* across a range of biologically meaningful perturbations, (2) estimated the rate of increase in targeted vital rates that would be enough for *D*. *waianaeensis* to persist following the reduction in abundance of an invasive rodent and non-native molluscs, and (3) quantified the economic cost and efficiency to suppress the invasive rodent and non-native molluscs.

## Results

**Figure 1:**
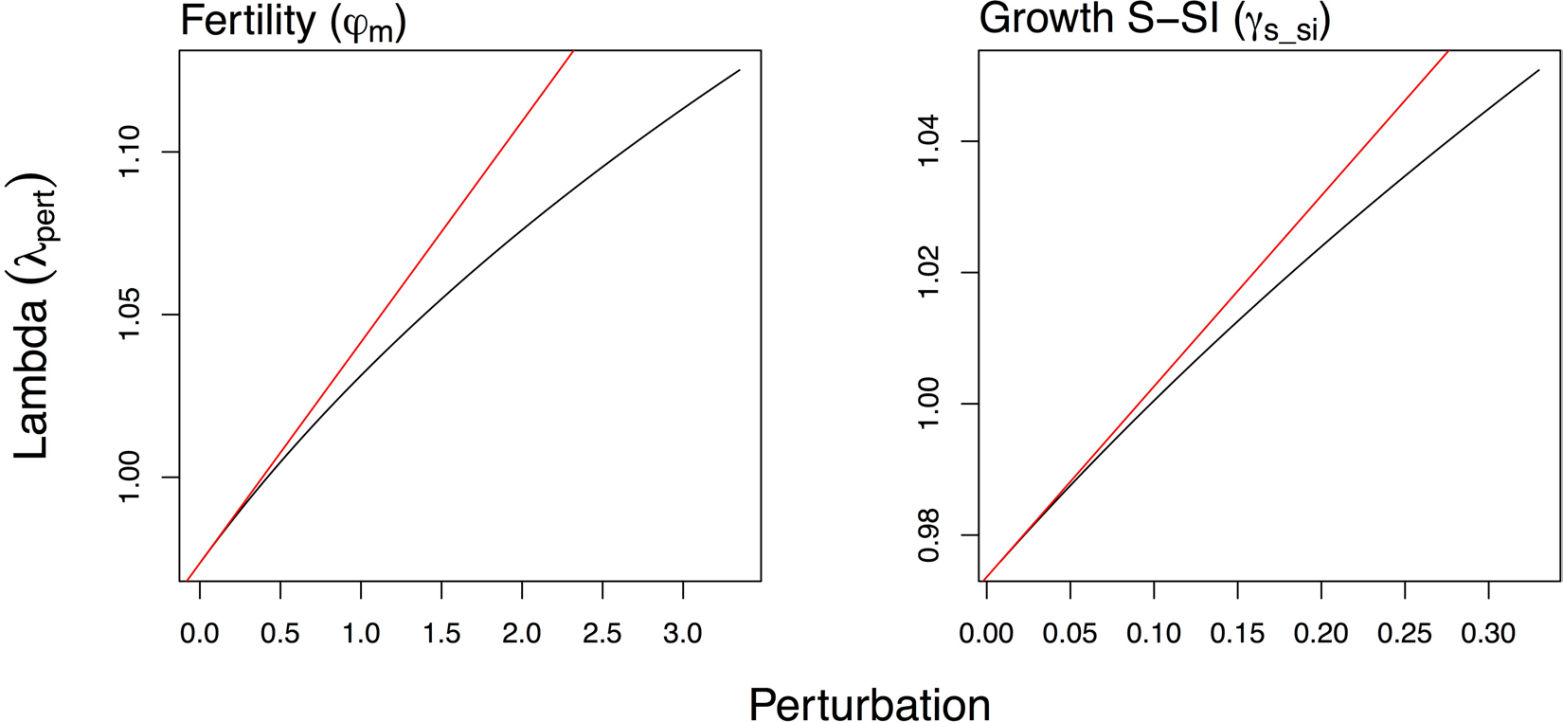
Transfer function analysis, where the black line illustrates the change in population growth rate across a range of biologically meaningful vital rate perturbations. The red line represents the slope of *λ* predicted from sensitivity. The vital rates are fertility (${\phi }_{{\rm{m}}}$) and seedling growth (*γ*_s−si_).

**Figure 2:**
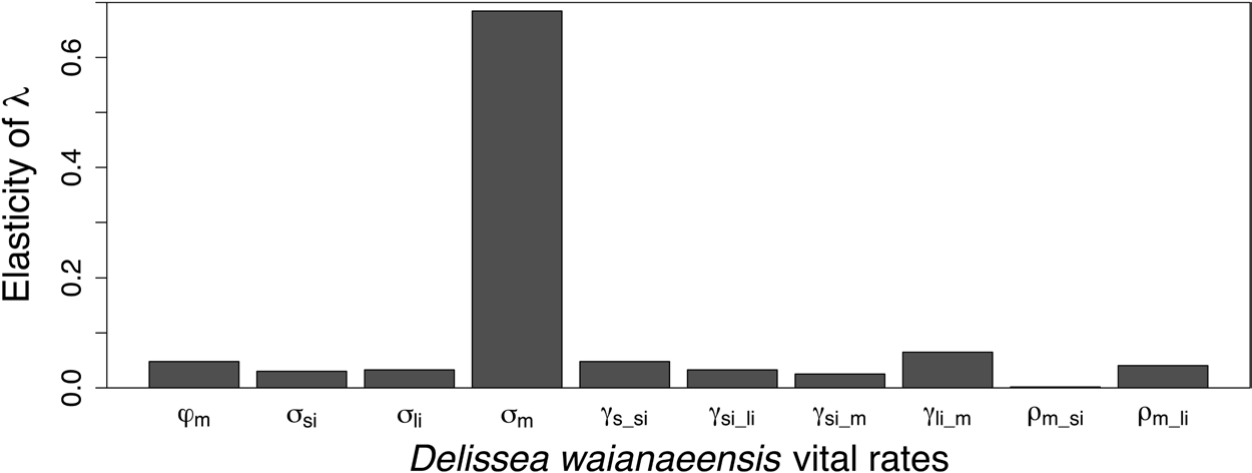
Elasticity analysis, which measures proportional sensitivity and is commonly used to assess how small perturbations to vital rates influence population growth rate. The vital rates are fertility ($\phi$), survival (*σ*), growth (*γ*), and shrinkage ($\rho$). The life stages are seedling (s), small immature (si), large immature (li), and mature (m).

**Figure 3:**
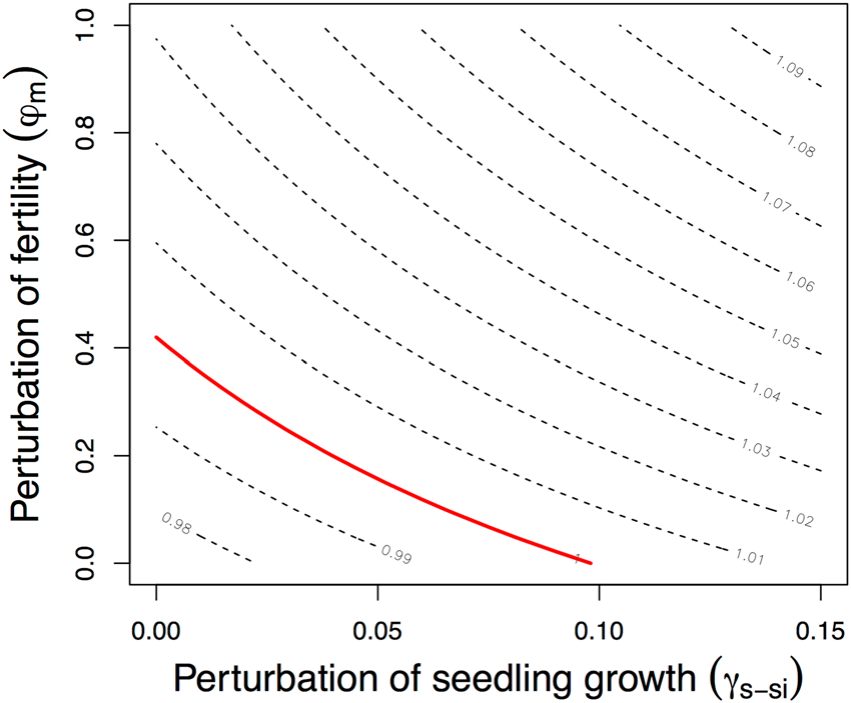
Transfer function analysis, which was used to identify the combinations of increases in fertility and seedling growth that would shift the population growth rate from declining to stable. The red line represents a population growth rate *λ*_*pert*_ = 1.

To assess the ecological and economical effects of invasive rodents and non-native molluscs on the population dynamics, we used a combination of transfer function analysis and economic cost analysis. Our results demonstrate that the effects of vital rate perturbations on *D*. *waianaeensis* population growth rate were nonlinear across a range of biologically meaningful perturbations (Fig. 1 and Appendix S1, Figure S1.1). Specifically, responsiveness of the population growth rate decreased as the magnitude of perturbation in fertility, growth of seedlings, and shrinkage of mature plants increased (Figure S1.1). Conversely, responsiveness of the population growth rate increased as the magnitude of perturbation in the stasis of all life stages and the growth of small vegetative plants increased (Figure S1.1). Considering only small perturbations (i.e., 0.01 change), we found that the survival of mature plants would have the greatest effect on population dynamics (Fig. 2). However, the survival of mature *D*. *waianaeensis* individuals was relatively high (86%) and was not a vital rate that could be improved by management (Appendix S1, Figure S1.3). The two vital rates that could be improved by management, fertility and seedling growth, had the same elasticity value (Fig. 2). However, a substantially larger increase in fertility (0.419) than in seedling growth (0.098) was required to shift the population growth rate from declining to stable (i.e., *λ* = 1, Fig. 3). There were also multiple combinations of increases in seedling growth and fertility that could achieve the desired outcome (Fig. 3). Using transfer function analysis, we found that increases in fertility and seedling growth following threat control translated to an increase in population growth rate from 0.97 to 1.125 and from 0.97 to 1.051, respectfully. The relative marginal efficiency *x* of increasing fertility ${{\epsilon }}_{f}$ and seedling growth ${{\epsilon }}_{s}$ on population growth rate was 2.706 (*i*.*e*., $x={{\epsilon }}_{f}/{{\epsilon }}_{s}$), which indicates the suppression of rodents was more economically efficient than the suppression of molluscs.

## Discussion

The long-term persistence of reintroduced populations is alarmingly low^2^. Thus, more threat management is necessary post-reintroduction to ensure the long-term persistence of most reintroduced species. In this context, it is critical that conservation biologists identify cost-effective combinations of restoration actions. Managing rare plant species native to oceanic islands is particularly challenging, as these species are typically threatened by numerous factors, some of which are more difficult to manage than others^12^. We emphasis that caution should be taking in using deterministic long-term projections, such as *λ* = 1, as a metric of restoration “success”. A more appropriate tool to evaluate population persistence is stochastic short-term dynamics (e.g., 10 year projections). However, as a comparative tool, deterministic long-term models are useful for prioritizing management actions. In the present study, we used transfer function analysis and relative marginal efficiency to establish biologically meaningful and economically efficient post-reintroduction strategies for a long-lived shrub in Hawaii. This study contributes to a growing body of literature that finds that large perturbations to fertility and earlier life stages can have smaller effects on population growth rate of long-lived species than would be expected based on elasticity analysis, which considers small perturbations of vital rates^9^.

Ignoring the biological limits in targeted vital rates when prioritizing restoration actions can also lead to ineffective management^13^. For example, Lubben, *et al*.^13^ found that management recommendations based solely on elasticity analysis, which ignores biological limits, would indicate conservation biologists should focus on increasing the survival of adult Serengeti cheetah. However, when the magnitude of change in targeted vital rates was considered, the authors found that multiple vital rates needed to be increased in order for Serengeti cheetah to persist, including the survival of adult and newborn cubs. For *D*. *waianaeensis*, it was critical to consider biological limits of vital rates perturbations to identify what part of the life cycle would have the greatest impact on population dynamics. Specifically, a greater increase in fertility (0.419) than in seedling growth (0.098) was needed to stabilize this reintroduced population (Fig. 3). Thus, considering only the magnitude of change in vital rates on population dynamics (i.e., cost negligent model), our results would suggest management should focus on improving seedling growth.

Seed consumption by rodents is an important threat to many rare species, especially for systems in which rodents are introduced^14–16^, or occur at unnaturally high densities due to human modification of landscapes^17,18^. Similarly, seedling herbivory by molluscs poses a significant threat to native oceanic island plants, especially Campanulaceae species such as *D*. *waianaeensis*^12,19^. In this study, we found that management recommendations based on the results of cost-negligent model would suggest conservation biologists should focus on increasing seedling growth by suppressing molluscs. However, improving fertility by reducing fruit consumption by rodents would be more cost effective. There are several reasons for the higher economic efficiency of rodent control than mollusc control. First, there are large differences in management-induced changes in targeted vital rates following threat control. While there can be a 5.9 fold increase in fertility following the suppression of rodents, seedling growth can only increase by up to 33% following the suppression of molluscs^12^. Secondly, it is less expensive to suppress rodents than it is to suppress molluscs (Appendix SI). The lower cost of rodent suppression than mollusc suppression is due, in part, to the shorter duration of time that rodents need to be suppressed (Appendix SI). While rodents only need to be suppressed during the *D*. *waianaeensis* fruiting season, molluscs need to be suppressed year round. Furthermore, technological advancements, such as the development of the self-resetting Goodnature A24 rodent traps, have improved the efficiency and reduced the labor hours needed to suppress rodents^20^. Similar technological advancements in mollusc control have not been achieved and should be a focus of future applied research and policy considerations.

Numerous studies examined the effects of invasive pests on the demography of rare species^21^. Surprisingly, to our knowledge, only two other studies have directly linked the economic cost of targeted threat control actions to changes in the population growth rate of managed species^10,11^. These studies also found that incorporating the costs of targeted restoration explicitly in demographic models resulted in optimal management recommendations that diverged from the cost-negligent managed recommendations. For example, Baxter, *et al*.^10^ found that elasticity analysis would focus on increasing the survival of the endangered Australian Helmeted Honeyeater, whereas cost-efficient management recommends would focus on increasing fecundity by reducing nest predation. The time is ripe for more demographic analyses that explicitly incorporate the cost of management actions for conservation planning. Many rare plant and animal populations have detailed demographic data and face multiple threats. In some places, such as Hawaii, approximate estimates for the economic costs of management are also readily available.

This study provides an example of how to develop efficient and effective management strategies for declining populations. Specifically, our study demonstrates how transfer function analysis can be used to set biologically meaningful increases in targeted life-stage transitions (i.e., matrix elements) that would be needed to reach a predefined restoration goal (e.g., population growth rate *λ* ≥ 1) and to incorporate several management strategies. Further, when multiple management strategies could likely be used to reach the desired restoration outcome, our results illustrate the importance of incorporating the cost of targeted threat control actions in demographic models in order to optimize management efficiency. Considering the limited financial resources allocated to conservation and the continual increase in the listing of rare and at-risk species^22^, using demographic models to identify the most economically efficient restoration strategy is becoming increasing desirable.

## Methods

### Study system

*Delissea waianaeensis* is a single or branched O’ahu endemic shrub typically reaching 1–3 m in height^23^. The fleshy fruit is an ovoid berry, with seeds that are 1.0–1.2 mm long and 0.4–0.6 mm wide^24^. Seed viability is relatively high, with a 95% mean germination rate^25^. The fleshy fruit is indicative of frugivorous bird dispersal^26^.

We studied the demography of *D*. *waianaeensis* in the Central Kaluaa gulch of the Honouliuli Forest Reserve, in the northern Wai’anae Mountains of O’ahu (HON; 21°28′N, −158°6′W). This *D*. *waianaeensis* population is a multi-year reintroduction effort, which has been actively managed for over a decade by the Nature Conservancy and the O’ahu Army Natural Resources Program (OANRP). Prior to plant reintroduction, an ecosystem level fence was constructed and feral ungulates were removed. At the site, there is also an ongoing suppression of invasive vegetation (for details see Bialic-Murphy *et al*., submitted). The two remaining biotic stressors faced by *D*. *waianaeensis* population are fruit-consuming black ship rat (*Rattus rattus*) and seedling-consuming non-native molluscs (Appendix S1, A). Both of these stressors are extremely disruptive to oceanic island ecosystems and are drivers of species decline and extinction^14,19^.

### Data collection and matrix construction

The life cycle of *D*. *waianaeensis* was categorized into four life stages based on height to the apical meristem: seedling (<2 cm), small immature (2 cm–35 cm), large immature (>35 cm and non-reproductive), and reproductively mature individuals (>35 cm). From 2010–2015, a total of 597 plants were permanently tagged and demographic data were collected annually in January–February. For each tagged plant we recorded survival, growth to the apical meristem, and reproduction (i.e., vegetative, flowering, or fruiting). Using these demographic data from 2010–2015, we constructed a mean 4 × 4 Lefkovitch transition matrix **A** (Caswell 2001):

$${\bf{A}}=(\begin{array}{cccc}0 & 0 & 0 & {\phi }_{m}\\ {\gamma }_{{\rm{s}}-{\rm{si}}} & {\sigma }_{{\rm{si}}} & 0 & {{\rm{\rho }}}_{{\rm{m}}\_{\rm{si}}}\\ 0 & {\gamma }_{{\rm{si}}-{\rm{li}}} & {\sigma }_{{\rm{li}}} & {{\rm{\rho }}}_{{\rm{m}}\_{\rm{li}}}\\ 0 & {\gamma }_{{\rm{si}}-{\rm{m}}} & {\gamma }_{{\rm{li}}-{\rm{m}}} & {\sigma }_{{\rm{m}}}\end{array})$$

The transition matrix **A** captures the probability of stasis *σ* the probability of survival and growth to the next life stage *γ*, the probability of shrinkage to the previous life stage ${\rm{\rho }}$, and seedling recruitment ${\phi }_{m}$ in the following discrete life stages: seedling (s), small immature (si), large immature (li), and reproductively mature (m). The term ${\phi }_{{\rm{m}}}$ is the mean total number of seedlings produced at time *t* + 1 by the total number of reproductively mature plants at time *t*. Since we had an additional year of data for fertility, the ${\phi }_{{\rm{m}}}$ term of matrix **A** is the mean fertility over six consecutive years (2009–2015). For modeling purposes, we assumed *D*. *waianaeensis* did not have a persistent seed bank. Although this assumption may influence long-term growth rate projections, it would not change the relative important of restoration strategies. We calculated the population growth rate of *D*. *waianaeensis* as the dominant eigenvalue, *λ*, of matrix **A**. We analyzed the sensitivity of *λ* to perturbations in matrix elements, and elasticity analysis (i.e., proportional sensitivity) following equations in^27^.

### Transfer function analysis

The exact relationship between the magnitude of change (δ) in vital rates and the population growth rate *λ* is given by^9^:

1 $${\delta }^{-1}={{\bf{c}}}^{{\rm{T}}}{({\lambda }_{pert}{\bf{I}}-{\bf{A}})}^{-1}{\bf{d}}$$

where **A** represents the transition matrix and **I** is an identity matrix. The terms **c** and **d** represent row and column vectors that determine the specific vital rates that will be perturbed. The term $\delta$ denotes the magnitude of the perturbation. We used eqn 1 to quantify the response of population growth rate *λ*_*pert*_ to a range of biologically meaningful perturbations $\delta$^28^, using the *popdemo* package (Stott *et al*. 2012b) in R version 3.1.0. We specifically tested the effects of biologically meaningful (a) increases in fertility ${\phi }_{m}$ following the suppression of *R*. *rattus*, and (b) increases in seedling growth *γ*_*s*_ following the suppression of non-native molluscs on *λ*_*pert*_. We also identified the magnitude of perturbation $\delta$ for fertility ${\phi }_{m}$ and seedling growth *γ*_*s*_ that was needed to reach a stable population growth rate, *λ*_*pert*_ = 1^29^. The range of biologically meaningful increases in fertility and seedling growth following threat control were determined using a combination of field experiments and the results of previous studies (Appendix S1, B).

### Relative marginal efficiency

We calculated the marginal efficiency to suppress non-native molluscs or *R*. *rattus*, following^10^:

2 $${{\epsilon }}_{k}=\frac{\partial \lambda }{\partial {C}_{k}}\,$$

where $\partial {C}_{k}\,$is the change in cost for achieving a management action *k*(i.e., increase in targeted vital rate) and $\partial \lambda$ represents the change in the population growth rate following investment in management *k*. The later was calculated using eqn 1. The efficiency of two manageme*n*t actions, *n* and *m*, can be estimated by calculating the relative marginal efficiency $x={{\epsilon }}_{n}/{{\epsilon }}_{m}$. If the relative marginal efficiency *x* is >1, then management action *n* is more efficient than *m*.

The costs to suppress *R*. *rattus* C_*f*_ and molluscs *C*_*s*_ were derived from the OANRP database and represent the average cost to suppress *R*. *rattus* and molluscs at other managed sites of comparable size to the *D*. *waianaeensis* site (Appendix S1, C). The suppression of *R*. *rattus* and molluscs both require an investment in variable costs (e.g., wages). The suppression of *R*. *rattus* also requires an upfront investment in fixed costs (e.g., equipment). To incorporate the cost of equipment needed to suppress *R*. *rattus* on a yearly basis, the fixed costs (e.g., equipment) were amortized over the lifespan of the equipment^30^. The total cost to suppress *R*. *rattus C*_*f*_ was calculated as the sum of the fixed and variable annual costs (Appendix S1, C).

### Data availability

The mean yearly transition matrix used to simulate the population dynamics for this study is reported in Appendix Figure S1.2 and deposited on the COMPADRE Plant Matrix Database.

## Electronic supplementary material

Appendix S1
