# Supplementary material for: Using Transfer Function Analysis to develop biologically and economically efficient restoration strategies
Source: Sci Rep. 2018 Feb 1;8:2094. doi: 10.1038/s41598-018-20178-7 (PMC5794749; doi:10.1038/s41598-018-20178-7)
Supplement: Supplementary file 1 — Appendix S1 [file 41598_2018_20178_MOESM1_ESM.pdf]

Supporting information for

Using Transfer Function Analysis to develop biologically and economically efficient restoration strategies

Authors: Lalasia Bialic-Murphy[1,2]*, Orou G. Gaoue[1,2,3,4], and Tiffany Knight [5,6,7]

[1] *Department of Botany, University of Hawai'i at Mānoa, Honolulu, Hawai'i , USA.*
[2] *Department of Ecology and Evolutionary Biology, University of Tennessee Knoxville, Knoxville, Tennessee, USA.*
[3] *Faculty of Agronomy, University of Parakou, Parakou, Benin.*
[4] *Department of Geography, Environmental Management and Energy Studies, University of Johannesburg, APK Campus, Johannesburg, South Africa*
[5] *Department of Community Ecology, UFZ, Helmholtz Centre for Environmental Research, Halle, Germany.*
[6] *Institute of Biology/Geobotany and Botanical Garden, Martin-Luther-University Halle-Wittenberg.*
[7] *German Centre for Integrative Biodiversity Research (iDiv) Halle-Jena-Leipzig, Leipzig, Germany.*

*Correspondence author: +1 808 443 7484, e-mail: lalasia.murphy@gmail.com

Appendix S1

A: Background information for *Rattus rattus* and non-native molluscs

Rattus rattus (black ship rat) is one of the most disruptive vertebrates to invade oceanic islands and often listed as a primary driver of population decline and extinction of native plants [1,2]. The estimated home range of *R. rattus* is 4 ha [3]. When foraging, *R. rattus* are the most active in areas with thick understory vegetation cover 10–30 cm in height [3]. *Rattus rattus* dens are often below ground in soil and fractured rock substrate, under logs, in thick understory vegetation, and inside partially dead trees [3]. Thought *R. rattus* are omnivores, seeds and fruits are the dominant portion of their diet [2]. Following consumption and digestion by *R. rattus*, small seeds (0.5–1.2 mm) remain intact and viable [4]. The relatively small size of *D. waianaeensis* seeds (1.0–1.2 mm) suggests that *R. rattus* do not alter seed viability of this taxon following consumption and digestion. However, the large home range, den characteristics, and foraging behavior of *R. rattus* imply *D. waianaeensis* seeds consumed by *R. rattus* are deposited in unsuitable habitat for seedling establishment.

The establishment of non-native molluscs (Mollusca: Gastropoda) is often implicated in the decline of oceanic island species [5,6]. Molluscs are generalist herbivores, primarily consuming foliage on the forest floor. In Hawaii, a total of 12 different non-native mollusc species have established throughout natural areas [7,8]. In mesic to wet forest communities in Hawaii, terrestrial molluscs significantly reduce the density of numerous native seedlings and thus, the suppression of non-native molluscs is often incorporated as part of the recovery strategy for endangered species [6,9]. Over the five-year

study period, we did not observe mollusc herbivory damage on later life stages. Thus, for modeling purposes, we assumed mollusc herbivory only influenced seedling survival and growth.

B: Range of biologically meaningful perturbations in targeted vital rates

The range of biologically meaningful perturbations that we used for this study was determined using a combination of field experiments and the results of previous studies. To set a realistic range of increases in fertility φ_m following the suppression of *R. rattus*, we quantified the percent of fruits consumed and the identity of the consumer using a modified version of the methods developed by Pender, et al. [10]. Given there are other potential frugivores that may consume *D. waianaeensis* fruits, including native and non-native birds, tracking tunnels were used to isolate the effects of *R. rattus* on fertility. Specifically, we installed 24 tracking tunnels at equal distance along four transects (50 cm x 10 cm x 10 cm; Connovation Limited, Auckland, New Zealand), with tracking cards inserted (The Black Trakka Gotcha Traps LTD, Warkworth, New Zealand) (Figure S1.1). The four transects spanned the length of the *D. waianaeensis* population; capturing intrapopulation habitat heterogeneity. Each tracking tunnel was baiting with one mature fruit and checked at a two-day interval. On each visit, fruit consumption was recorded and the tracking tunnels were re-baited. The footprints left on the tracking cards each visit were used to identify the frugivore consuming *D. waianaeensis* fruits (Figure S1.1). In total, the tracking tunnels were baited five times during the 2015 fruiting season. In this field experiment, we found that the mean *R. rattus* frugivory rate was 83%. To mimic the effects of *R. rattus* control on fertility and population dynamics, we set the

biologically feasible increases in φ_m from the observed value to a 5.9 fold increase. The proportion of fruits consumed by *R. rattus* at the *D. waianaeensis* population was consistent with previous studies that have examined the effect of *R. rattus* on the fertility of a related taxon in the Campanulaceae group, *Cyanea superba* ssp. *superba* [10]. Though it is possible that rodent control increases the effects of other stressors on plant vital rates, this has not been documented at similar field sites in Hawaii following rodent suppression [10].

To set a range of biologically meaningful increases in seedling growth $\gamma_{s\text{-}si}$ following the suppression of non-native molluscs, we used the results of a previous field experiment [9]. In this experiment, 200 seeds of three localized Hawaiian endemic species (*Cyanea superba* ssp. *superba, Cyrtandra dentata, and Schiedea obovata*) were sown on the top layer of soil in 12 plots, 15m x 15m in diameter. Six plots were treated with a molluscicide, Sluggo (Neudorff Co., Fresno, California), and the other six plots were left exposed to normal herbivory intensity at the field site. The density of seedlings in each plot was recorded on a weekly basis for six weeks. This study illustrated that seedling density between the mollusc suppression and mollusc control treatments were significantly different for localized endemic plants, by up to 33% [9]. The range of biologically feasible increases in $\gamma_{s\text{-}si}$ following the suppression of non-native molluscs that we used for our study was from the observed $\gamma_{s\text{-}si}$ value to $\gamma_{s\text{-}si}$ +.33 by a perturbation magnitude of .01.

C: Cost of management actions

To suppress *R. rattus* at the *D. waianaeensis* field site would require 20 Goodnature A24 self-resetting multi-species kill traps (Tyler Bogardus, personal communication). The per unit cost of the Goodnature A24 self-resetting multi-species kill trap was $125 and the lifespan of the traps was 10–15 years (Tyler Bogardus, personal communication). The total field time needed to setup the *R. rattus* trap grid and maintain it over one *D. waianaeensis* fruiting season was 60 hours ($25.92 per hour x 60 hours = $1,555.2; Oahu Army Natural Resources Program, unpublished data). The total yearly field time needed to suppress molluscs was 176 hours ($25.92 per hour x 176 hours = $4,562; Oahu Army Natural Resources Program, unpublished data). The lower total field time needed to suppress *R. rattus* than to suppress molluscs is due, in part, to the shorter duration of time that *R. rattus* needs to be suppresses. While *R. rattus* only needs to be suppressed during the *D. waianaeensis* fruiting season, molluscs need to be suppressed year round. The fixed cost of equipment to suppress *R. rattus* was $2,500 (i.e., 20 Goodnature A24 rodent traps x $125 per trap). However, when amortized over the lifetime of the equipment (i.e., 10 years), the yearly equipment cost to suppress *R. rattus* was $250. Including labor costs, the total yearly cost to suppress *R. rattus* (i.e., yearly fixed cost of equipment and labor costs) was $1,805. For mollusc suppression, there is no upfront fixed cost of equipment and the total yearly labor cost was $4,562.

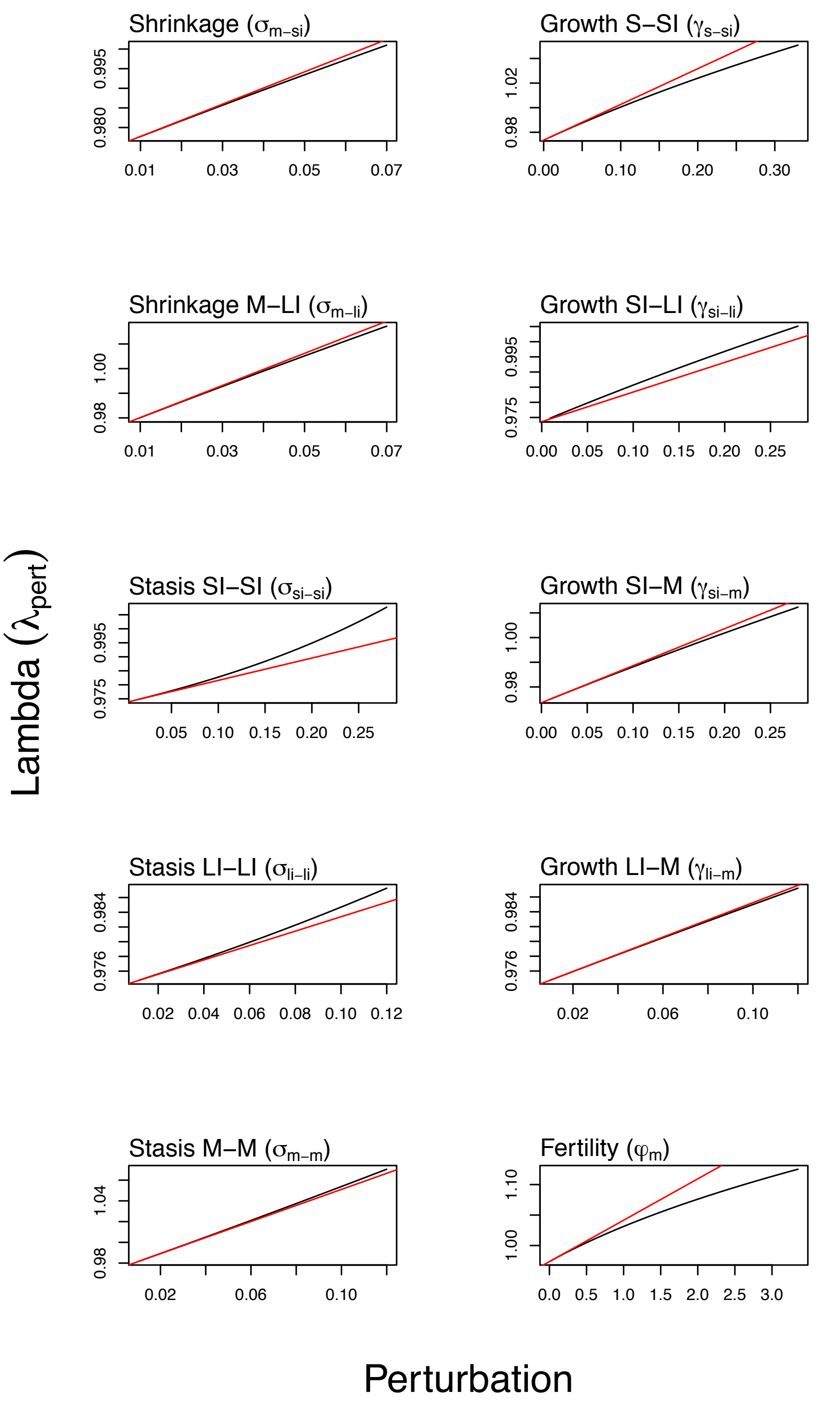

Figure S1.1: Transfer function analysis, where the black line illustrates the change in population growth rate across a range of vital rate perturbations. The red line represents the slope of λ predicted from sensitivity. The vital rates are fertility (φ), survival (σ), growth (γ), and shrinkage (ρ). The life stages are seedling (s), small immature (si), large immature (li), and mature (m).

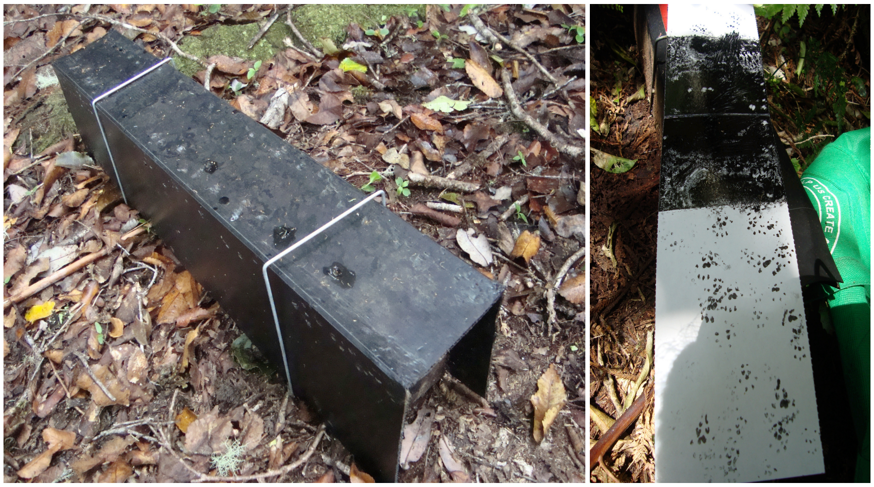

Figure S1.2: The image to the left is a photo of the tracking tunnels (50 cm x 10 cm x 10 cm; Connovation Limited, Auckland, New Zealand) used to quantify fruit consumption by *Rattus rattus*. The image to the right is a tracking card (The Black Trakka Gotcha Traps LTD, Warkworth, New Zealand) with footprints of *Rattus rattus* following fruit consumption.

	s	si	li	m
s	0.000	0.000	0.000	0.687
si	0.161	0.368	0.000	0.004
li	0.000	0.188	0.326	0.061
m	0.000	0.163	0.544	0.860

Figure S1.3: Mean yearly transition matrix for of *Delissea waianaeensis.* The life stages are life stages are seedling (s); small immature > 35 cm (si); large immature, >35 cm (li), and mature signs of reproduction (m).

References:

1 Towns, D. R., Atkinson, I. A. & Daugherty, C. H. Have the harmful effects of introduced rats on islands been exaggerated? *Biological invasions* **8**, 863-891 (2006).

2 Shiels, A. B., Pitt, W. C., Sugihara, R. T. & Witmer, G. W. Biology and impacts of pacific island invasive species. 11. *Rattus rattus*, the Black Rat (Rodentia: Muridae). *Pacific Science* **68**, 145-184 (2014).

3 Shiels, A. B. *Ecology and impacts of introduced rodents (Rattus spp. and Mus musculus) in the Hawaiian islands* 72 thesis, Dissertations & Theses @ University of Hawai`i at Manoa. (860957214), (2010).

4 Shiels, A. B. & Drake, D. R. Are introduced rats (Rattus rattus) both seed predators and dispersers in Hawaii? *Biological Invasions* **13**, 883-894 (2011).

5 Cowie, R. H., Dillon Jr, R. T., Robinson, D. G. & Smith, J. W. Alien non-marine snails and slugs of priority quarantine importance in the United States: a preliminary risk assessment. *American Malacological Bulletin* **27**, 113-132 (2009).

6 Joe, S. M. & Daehler, C. C. Invasive slugs as under-appreciated obstacles to rare plant restoration: evidence from the Hawaiian Islands. *Biological Invasions* **10**, 245-255, doi:10.1007/s10530-007-9126-9 (2007).

7 Cowie, R. New records of alien nonmarine mollusks in the Hawaiian Islands. *Bishop Museum Occasional Papers* **59**, 48-50 (1999).

8 Cowie, R. H. Catalog and bibliography of the nonindigenous nonmarine snails and slugs of the Hawaiian Islands. *Bishop Museum Occasional Papers*, 1-66 (1997).

9 Kawelo, H. K., Harbin, S. C., Joe, S. M., Keir, M. J. & Weisenberger, L. in *Plant Reintroduction in a Changing Climate* 209-226 (Springer, 2012).

10 Pender, R. J., Shiels, A. B., Bialic-Murphy, L. & Mosher, S. M. Large-scale rodent control reduces pre-and post-dispersal seed predation of the endangered Hawaiian lobeliad, Cyanea superba subsp. superba (Campanulaceae). *Biological invasions* **15**, 213-223 (2013).
